# Pulse duration settings in subthalamic stimulation for Parkinson's disease

**DOI:** 10.1002/mds.27238

**Published:** 2017-11-22

**Authors:** Frank Steigerwald, Lars Timmermann, Andrea Kühn, Alfons Schnitzler, Martin M. Reich, Anna Dalal Kirsch, Michael Thomas Barbe, Veerle Visser‐Vandewalle, Julius Hübl, Christoph van Riesen, Stefan Jun Groiss, Alexia‐Sabine Moldovan, Sherry Lin, Stephen Carcieri, Ljubomir Manola, Jens Volkmann

**Affiliations:** ^1^ Department of Neurology Universitätsklinikum Würzburg Würzburg Germany; ^2^ Departments of Neurology and Functional & Stereotactic Neurosurgery Universitätsklinikum Köln Köln Germany; ^3^ Department of Neurology, Charité University Medicine Berlin Germany; ^4^ Department of Neurology Universitätsklinikum Düsseldorf Düsseldorf Germany; ^5^ Boston Scientific Neuromodulation Valencia California USA; ^6^ Boston Scientific Neuromodulation Diegem Belgium

**Keywords:** Deep brain stimulation, Parkinson's disease, pulse width, stimulation parameters, subthalamic

## Abstract

**Background:**

Stimulation parameters in deep brain stimulation (DBS) of the subthalamic nucleus for Parkinson's disease (PD) are rarely tested in double‐blind conditions. Evidence‐based recommendations on optimal stimulator settings are needed. Results from the CUSTOM‐DBS study are reported, comparing 2 pulse durations.

**Methods:**

A total of 15 patients were programmed using a pulse width of 30 µs (test) or 60 µs (control). Efficacy and side‐effect thresholds and unified PD rating scale (UPDRS) III were measured in meds‐off (primary outcome). The therapeutic window was the difference between patients’ efficacy and side effect thresholds.

**Results:**

The therapeutic window was significantly larger at 30 µs than 60 µs (*P* = ·0009) and the efficacy (UPDRS III score) was noninferior (*P* = .00008).

**Interpretation:**

Subthalamic neurostimulation at 30 µs versus 60 µs pulse width is equally effective on PD motor signs, is more energy efficient, and has less likelihood of stimulation‐related side effects. © 2017 The Authors. Movement Disorders published by Wiley Periodicals, Inc. on behalf of International Parkinson and Movement Disorder Society.

The efficacy and side effect profile of deep brain stimulation (DBS) critically depends on the electrical activation of specific neural elements within a small brain volume.^1^ Factors that determine the volume of tissue activated (eg, electrode position, electrical field shaping effects of contact selection, polarity, and current amplitude) affect the stimulation outcome. Neurostimulation effects also depend on the temporal characteristics of the stimulus waveform, and changes in pulse duration may impact the selectivity of neural activation within a given stimulation volume.^2^

The CUSTOM‐DBS study was designed to address the need for an evidence‐based update of DBS programming guidelines,^1^ including the expanded parameter space of novel DBS devices. The primary outcome was the use of shorter pulse widths than 60 µs to expand the window between thresholds for therapeutic effects and side effects, without compromising efficacy, and for which there is a well‐understood and plausible mechanism of action.^3^ An exploratory outcome was the impact of current fractionalization among adjacent contacts using multiple independent current control. Here, we report results on the pulse duration programming change between 30 and 60 µs in an acute, double‐blind comparison. The results of current steering are included in the Supplementary Appendix.

## Methods

### Participants

PD patients bilaterally implanted with a Vercise DBS system (Boston Scientific, Valencia, California) in the STN for at least 3 months were included at 4 German centers. All patients had a Unified Parkinson's Disease Rating Scale (UPDRS) subset III score of ≥30 in the preoperative meds‐off state, and ≥30% symptom reduction in the meds‐off state (UPDRS III score) by DBS. To ensure sufficient rigidity to reliably measure a threshold, a meds‐off rigidity score ≥ 2 was required in the evaluated arm (UPDRS III). Patients were excluded if severe tremor would interfere with measurements, defined as meds‐off resting and/or action tremor score ≥3 on the evaluated side (UPDRS III). Patient demographics are shown in Supplementary Table 1.

All sites obtained ethics committee approval. Written informed consent was given by all patients before study inclusion. The study is registered with ClinicalTrails.gov (NCT01896115).

### Randomization and Masking

Test conditions were administered in random order with masking of patients and the examiner to treatment assignment. Random permutations of the treatment sequence, stratified by site with a block size of 4, were pregenerated along with a set of mutually distinct identification codes. To mask the randomization assignment, each site only received the codes, each sealed within a numbered envelope to be opened sequentially as patients enrolled into the study. The nonblinded programmer controlled the pulse width and electrode settings according to the randomization sequence matched to the code using a computer positioned out of the examiner's sight.

### Procedures

Primary data collection occurred in a single programming visit. Patients arrived in the practically defined meds‐off state (at least 12‐hour drug withdrawal). The clinical DBS effect was washed out by turning the device off for at least 1 hour. Testing was started when the patient and examiner agreed that a representative off‐state was reached. DBS was then turned back on contralateral to the clinically more affected side of the body. To evaluate pulse‐width effects, cathodic stimulation was delivered through the best single therapeutic electrode (monopolar against generator case), fixed at 130 Hz; pulse width was randomly assigned to either test (30 µs) or control (60 µs) settings. At each setting, amplitude was gradually increased until a side effect threshold was measured (defined as the minimum amplitude for any side effect persisting after 2 minutes of stimulation). Rigidity was evaluated 0.1 mA below the side effect threshold to assess the maximum rigidity control possible at that setting. The efficacy threshold was defined as the amplitude at which rigidity reoccurred by gradually decreasing the stimulation in steps of 0.1 mA.

**Figure 1:**
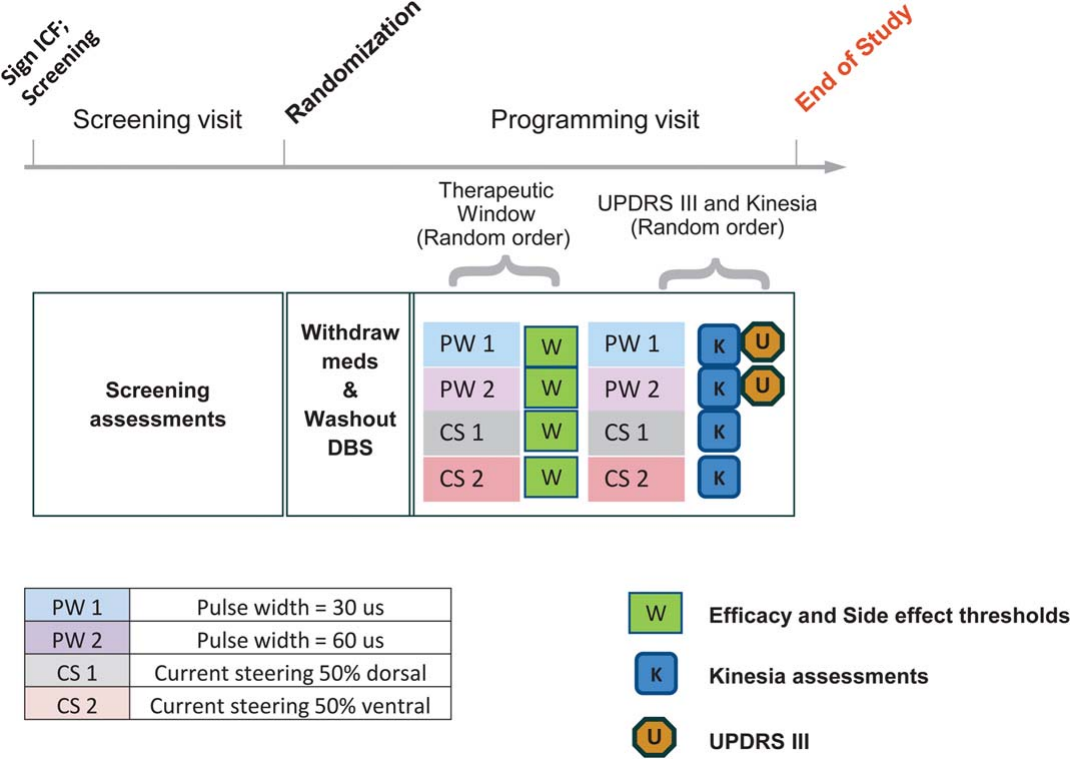
Schematic of the CUSTOM‐DBS study, a multicenter, double‐blind, randomized controlled trial. At the programming visit, the therapeutic window was first measured in a randomized, double‐blind assessment at four different program settings. Double‐blind UPDRS III and exploratory quantitative assessments (Kinesia assessments of rest tremor, finger tapping, and rapidly alternating movement tasks; Kinesia ProView, Great Lakes Neurotechnologies) were then taken at the efficacy threshold for rigidity for each setting. In addition to test and control pulse width settings, current steering settings were also tested as exploratory endpoints. Stimulation was activated on the best therapeutic contact as identified by clinical practice and programmed according to the figure. [Color figure can be viewed at wileyonlinelibrary.com]

Efficacy was measured in 2 ways after at least 15 minutes of continuous stimulation at the previously determined efficacy threshold for each setting: UPDRS III evaluation (including lateralized scores calculated by summing items for left and right sides separately), and an objective, quantitative assessment of tremor and bradykinesia using a finger‐worn motion sensor (Kinesia ProView, Great Lakes Neurotechnologies, Cleveland, Ohio). In the latter assessment, patients performed 3 tasks while wearing a motion sensor on the index fingertip: rest tremor assessment, finger‐tapping task, and rapidly alternating‐movement task. The study design is summarized in Fig 1.

Efficiency of neurostimulation was calculated as the total charge delivered per pulse (Q = I * pulse width) at the efficacy threshold.

### Statistical Analysis

The coprimary endpoints of this study were (1) superiority of the therapeutic window size and (2) noninferiority of stimulation efficacy (UPDRS III) on the most affected side of contralateral DBS with 30 µs compared to 60 µs pulse width. The 2 endpoints were each assessed using a 1‐sided paired *t* test at an alpha level of 0.025, applying the Bonferroni correction to preserve the family‐wise error rate. A margin of 5 points, representing the minimal clinically relevant difference,^4^ was established as the noninferiority margin for UPDRS III.

Sample size calculations suggested that the first coprimary endpoint (superiority of therapeutic window size) could be met after 16 participants, whereas the noninferiority endpoint would require 40 enrolled participants. Therefore an interim analysis was planned after enrollment of the first 16 patients and confirmed that both coprimary endpoints could be met at this stage. Therefore, the study could be discontinued, as per protocol, after the enrollment of 16 participants.

## Results

### Therapeutic Efficacy and Side Effect Thresholds

Of 16 enrolled participants, 1 did not tolerate reprogramming and withdrew before any data could be collected. Therefore, 15 patients completed the study.

**Figure 2:**
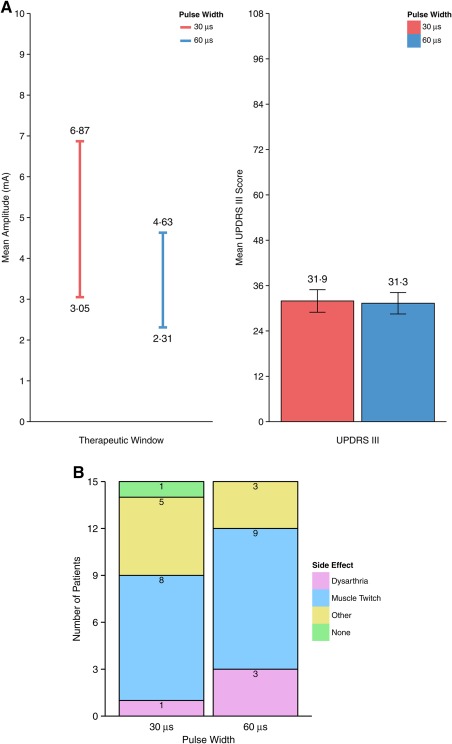
Coprimary endpoints. (**A**) Superiority of the therapeutic window size (left) and noninferiority of UPDRS III (right) at a short‐pulse width. Error bars represent ± 1 standard error. (**B**) Side effect observed at threshold during the therapeutic measurement. The most common side effects were dysarthria and muscle twitch related to pyramidal tract activation. [Color figure can be viewed at wileyonlinelibrary.com]

For these 15 participants, stimulation at 30 μs resulted in a significantly larger therapeutic window than 60 μs (mean 3.82 vs 2.32 mA; *P* = .0009; Fig 2A). The most common side effect thresholds were those for muscle contractions and dysarthria related to pyramidal tract activation (Fig. 2B). Efficacy at the rigidity threshold at 30 µs was noninferior to 60 µs as measured by total UPDRS motor score (mean 31.9 vs 31.3; *P* = .00008) or by lateralized motor score for the evaluated side (18.8 vs 18.1; *P* = .00051). Noninferiority of the stimulation efficacy at 30 µs was further confirmed by kinematic measures of rest tremor, finger‐tapping, or rapidly alternating movements (Supplementary Fig. 1).

### Efficiency of Neurostimulation

As predicted by the strength‐duration relationship^5^ efficacy and side effect thresholds were higher at a pulse width of 30 µs (3.05 mA and 6.87 mA, respectively) when compared with 60 µs (2.31 mA and 4.63 mA). However, neurostimulation efficiency is not measured by the threshold amplitude, but by the required amount of charge—a function of both amplitude and pulse width.^6^ The total charge delivered per pulse at efficacy threshold was lower at 30 µs pulse width, indicating that short pulse width stimulation is more efficient at exciting the neural elements responsible for therapeutic benefit (Supplementary Fig. 2; −34% difference; mean charge per pulse = 91.6 nC/pulse at 30 µs, 138.4 nC/pulse at 60 µs).

## Discussion

In DBS therapy, amplitude, pulse width, and frequency settings are adjusted in each patient individually, starting with standard values, to achieve the maximum reduction of clinical signs with minimal side effects and energy consumption.^6^ We have demonstrated here that standard programming settings used in STN‐DBS for PD may not be optimal.

When tested in a double‐blind, acute challenge, a shorter pulse width of 30 μs was superior to the standard 60 μs in terms of minimizing the likelihood of stimulation‐related side effects while maintaining the same therapeutic efficacy at a lower energy cost. Modelling data suggest that the mechanism of stimulation at short pulse duration may be best explained by focusing on excitation of smaller diameter axons near the electrode and a steeper falloff for activation of thick myelinated axons with increasing radius of current spread.^3^ This would reduce the likelihood of inadvertent stimulation of pyramidal tract fibers causing muscle contractions or dysarthria, the most frequently observed signs, when testing adverse effect thresholds in this study.

It should be noted that 30 μs itself may not be optimal. The programming time limitations prevented an exhaustive comparison of pulse width settings, and even shorter pulse widths might further expand the therapeutic window. In DBS, chronaxie for tremor suppression was previously estimated at approximately 65 to 75 µs,^7^ indicating that shorter pulse widths can excite the target fibers at safe current amplitudes. We selected 30 µs as the test pulse width based on a small pilot study^3^ because there was a steep increase of side effect thresholds at 20 µs often exceeding an arbitrarily chosen upper testing limit of 10 mA. In clinical practice, many patients might benefit from reducing pulse widths from conventionally used 60 µs or more to 30 to 50 µs, in cases where side effects do occur.

We were unable to explore other settings that may be superior. The number of combinations of pulse width, frequency, and voltage available within the recommended charge density limit was calculated to be 12,964 in older DBS devices,^6^ and since then that number of combinations has only increased with the introduction of additional electrodes, expanded pulse width range, current steering, and other programmable features of the device. It is not possible to test all settings in a single programming visit, and more studies are needed to define the optimal parameter space for target signs.

Another limitation was the lack of data on efficacy of chronic stimulation at a short pulse width. The participants were only exposed to each stimulation setting for a short time during a single programming visit; it is possible that long‐term stimulation would have revealed differences between short and conventional pulse widths that were not apparent during the acute visit. However, the blinded assessment of motor signs (UPDRS III) during an acute stimulation challenge has previously been used as the primary efficacy endpoint in DBS studies^8^, ^9^, ^10^ and reflected the chronic benefit of DBS.

Despite these limitations, few controlled studies are aimed at achieving optimization of DBS programming, and this is the first double‐blind assessment of the effect of a shorter pulse width and 1 of only a handful of DBS programming studies that have ever been conducted in a double‐blind condition.

In conclusion, stimulation using a shorter than currently recommended pulse width may be more efficient at achieving therapeutic efficacy and less likely to reach a side effect threshold. This may translate into a fundamentally new basic parameter setting for patients with DBS in PD.

## Author Roles

1) Research project: A. Conception, B. Organization, C. Execution; 2) Statistical Analysis: A. Design, B. Execution, C. Review and Critique; 3) Manuscript: A. Writing of the first draft, B. Review and Critique.

F.S.: 1B, 1C, 3A, 3B

L.T.: 1A, 1B, 1C, 3A, 3B

A.K.: 1B, 1C, 3A, 3B

A.S.: 1B, 1C, 3A, 3B

M.M.R.: 1B, 1C, 3A, 3B

A.D.K.: 1B, 1C, 3A, 3B

M.T.B.: 1B, 1C, 3A, 3B

V.V.‐V.: 1B, 1C, 3A, 3B

J.H.: 1B, 1C, 3A, 3B

C.R.: 1B, 1C, 3A, 3B

S.J.G.: 1B, 1C, 3A, 3B

A.‐S.M.: 1B, 1C, 3A, 3B

S.L.: 1B, 1C, 3A, 3B

S.C.: 1B, 1C, 3A, 3B

L.M.: 1B, 1C, 3A, 3B

J.V.: 1A, 1B, 1C, 3A, 3B

## Financial disclosures of all authors (for the preceding 12 months)

F.S. reports personal fees from Boston Scientific, personal fees from Medtronic, and personal fees from St. Jude Medical outside the submitted work. L.T. reports grants and personal fees from Bayer Healthcare, grants, personal fees and nonfinancial support from UCB Pharma GmbH (UCB) Schwarz Pharma, grants, personal fees and nonfinancial support from Archimedes Pharma, grants, personal fees and nonfinancial support from TEVA Pharma, grants, personal fees and non‐financial support from Lundbeck Pharma, personal fees from Medas Pharma, grants, personal fees and non‐financial support from Desitin Pharma, personal fees and non‐financial support from GlaxoSmithKline, personal fees and non‐financial support from Orion Pharma, grants, personal fees and non‐financial support from Abbvie, personal fees from TAD Pharma, grants from German Research Foundation, grants from German Ministry of Education and Health, grants from Manfred and Ursula Müller Foundation, grants from Klüh Foundation, grants from Hoffnungsbaum e.V., grants from Neurodegeneration with Brain Iron Accumulation Disorders Society USA, grants from Deutsche Parkinson Vereinigung, and grants from zur Rose Pharma outside the submitted work. A.K. reports other from Boston Scientific, grants and other from Medtronic, grants and other from St Jude Medical, other from Ipsen Pharam, outside the submitted work. A.S. reports personal fees from Medtronic, personal fees from St Jude Medical, personal fees from UCB, personal fees from MEDA Pharma GmbH Pharma, grants and personal fees from Teva Pharma, personal fees from GlaxoSmithKline, personal fees from Novartis, personal fees from Abbvie, outside the submitted work. M.M.R. has been a member of the advisor board of Medtronic; has received grant support from Boston Scientific, St. Jude and TEVA; and has received honoraria for speaking from Medtronic. A.D.K. reports grants from Abbvie, outside the submitted work. M.T.B. reports grants from Medtronic Inc., outside the submitted work. V.V.‐V. reports grants and non‐financial support from Medtronic, Boston Scientific, SAPIENS, and St. Jude Medical, and personal fees from Medtronic and St Jude Medical, as well as a grant and nonfinancial support from Boston Scientific outside the submitted work. S.J.G. reports personal fees from Boston Scientific, personal fees from Medtronic, and nonfinancial support from Actelion outside the submitted work. S.L., S.C., and L.M. report employment with Boston Scientific. J.V. reports grants and personal fees from Medtronic, personal fees from Allergan, personal fees from Merz, personal fees from UCB, personal fees from Abbvie, personal fees from TEVA, personal fees from Zambon, and personal fees from Bial outside the submitted work. J.H., C.R., A.‐S.M. have nothing to disclose.

## Supporting information

Additional Supporting Information may be found in the online version of this article at the publisher's website.

Supplementary Information Figure 1

Supplementary Information Figure 2

Supplementary Information Table 1

Supplementary Information 1

Supplementary Information 2
