# Supplementary material for: Pulse duration settings in subthalamic stimulation for Parkinson's disease
Source: Mov Disord. 2017 Nov 22;33(1):165–9. doi: 10.1002/mds.27238 (PMC5813170; doi:10.1002/mds.27238)

Supplementary Figure 1. Efficacy as measured by quantitative assessments of tremor and bradykinesia (Kinesia ProView, Great Lakes Neurotechnologies).

This assessment has been validated against clinician scores for tremor[1] and bradykinesia.[2] Kinesia scores for rest tremor (top), finger-tapping (middle), and Rapidly Alternating Movements (RAM) (bottom) were similar for the short pulse width and standard pulse width settings. All error bars represent +/- 1 standard error.

1 Giuffrida J, Riley D, Maddux B, Heldman D. Clinically deployable Kinesia technology for automated tremor assessment. Mov Disord 2009; 24(5): 723 -730.

2 Heldman D, Giuffrida J, Chen R, et al. The modified bradykinesia rating scale for Parkinson's disease: reliability and comparison with kinematic measures. Mov Disord 2011; 26(10): 1859 -1863.

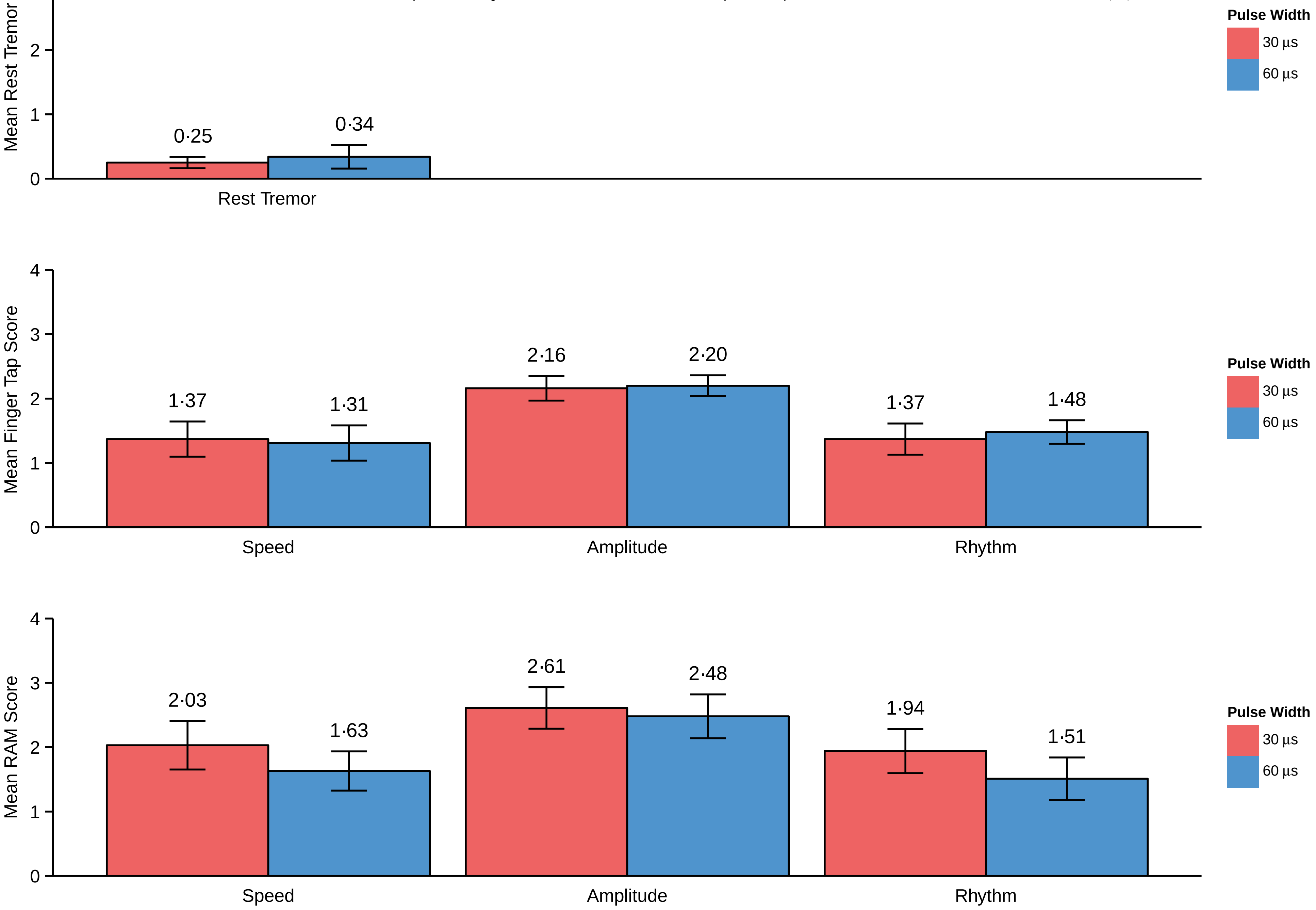

Supplement: Supplementary file 1 — Supplementary Information Figure 1 [file MDS-33-165-s001.pdf]
