# Supplementary material for: Pulse duration settings in subthalamic stimulation for Parkinson's disease
Source: Mov Disord. 2017 Nov 22;33(1):165–9. doi: 10.1002/mds.27238 (PMC5813170; doi:10.1002/mds.27238)
Supplement: Supplementary file 2 — Supplementary Information Figure 2 [file MDS-33-165-s002.pdf]

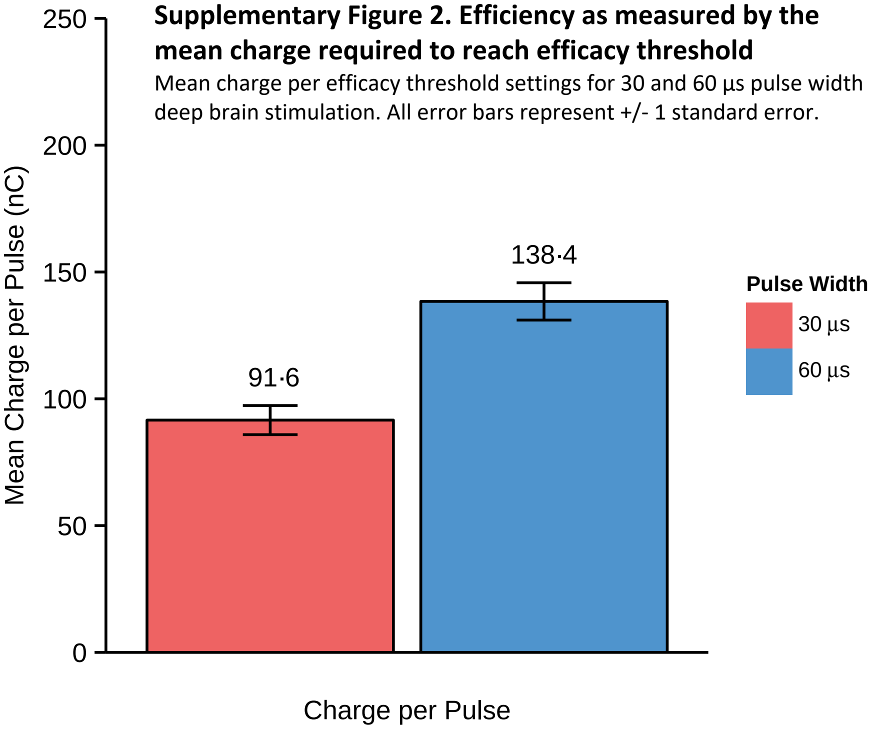

Supplementary Figure 2. Efficiency as measured by the mean charge required to reach efficacy threshold

Mean charge per efficacy threshold settings for 30 and 60 µs pulse width deep brain stimulation. All error bars represent +/- 1 standard error.
