# Supplementary material for: Pulse duration settings in subthalamic stimulation for Parkinson's disease
Source: Mov Disord. 2017 Nov 22;33(1):165–9. doi: 10.1002/mds.27238 (PMC5813170; doi:10.1002/mds.27238)
Supplement: Supplementary file 3 — Supplementary Information Table 1 [file MDS-33-165-s003.pdf]

Supplementary Table. Patient demographics.

	Mean (SD),<br>[min, max]
Age at informed consent, years	58·9 (7·7),<br>[47, 73]
Years taking Parkinson's medications	11·3 (4·0),<br>[5, 18]
Baseline UPDRS III<br>(*stim off*, *meds off*)	38·3 (8·4),<br>[27, 56]
Months post-DBS implant	16·9 (12·0),<br>[4·9, 39·5]
Gender, %	87 M, 13 F
Handedness, %	7 L, 93 R

DBS = deep brain stimulation; L = left; R = right; SD = standard deviation; UPDRS = unified Parkinson's disease rating scale
