# Supplementary material for: Pulse duration settings in subthalamic stimulation for Parkinson's disease
Source: Mov Disord. 2017 Nov 22;33(1):165–9. doi: 10.1002/mds.27238 (PMC5813170; doi:10.1002/mds.27238)
Supplement: Supplementary file 4 — Supplementary Information 1 [file MDS-33-165-s004.pdf]

Pulse duration settings in subthalamic stimulation for Parkinson’s disease

Frank Steigerwald, MD[1], Lars Timmermann, MD[2], Andrea Kühn, MD[3], Alfons Schnitzler, MD[4], Martin M Reich, MD[1], Anna Dalal Kirsch, MD[1], Michael Thomas Barbe, MD[2], Veerle Visser-Vandewalle, MD[2], Julius Hübl, MD[3], Christoph van Riesen, MD[3], Stefan Jun Groiss, MD[4], Alexia-Sabine Moldovan, MD[4], Sherry Lin, PhD[5], Stephen Carcieri, PhD [5], Ljubomir Manola, PhD[6], Jens Volkmann, MD[1]

Supplementary material

The CUSTOM-DBS study was designed to address the need for an evidence-based update of DBS programming guidelines. The primary outcome was the use of shorter pulse widths than 60 µs. An exploratory outcome was the impact of current fractionalization among adjacent contacts using multiple independent current control. This supplement details the exploratory study outcomes related to current fractionalization, which could not be included in the main manuscript due to space restrictions.

Rationale. The use of multiple-source, constant-current stimulation may prevent the spread of the electrical field into adjacent fiber tracts related to side effects. Here, we report results using current steering in an acute, double blind comparison with stimulation on a single electrode at the conventional pulse width.

Please refer to the main manuscript for details on the study population, including inclusion and exclusion criteria.

Methods. Data collection occurred in the same programming visit, in which we also tested different pulse duration settings. The change in therapeutic window using current steering (with 50% of the current assigned to the ventral or dorsal adjacent electrode) was measured as an exploratory endpoint. We tested two “current steering settings”, where stimulation was fixed at 130 Hz and 60 µs pulse width and delivered through two electrodes simultaneously: the best single therapeutic electrode, and either the dorsal or ventral adjacent electrode.

All subjects were exposed to all four stimulator settings (two pulse width settings and two current steering settings) in random order. At each setting, amplitude was gradually increased until a side effect threshold could be measured. Side effect threshold was defined as the minimum amplitude for any limiting side effect persisting after two minutes of stimulation. Rigidity was then evaluated 0.1 mA below the side effect threshold to assess the maximum rigidity control possible at that setting. The efficacy threshold was then defined as the amplitude at which rigidity reoccurred by gradually decreasing the stimulation in steps of 0.1 mA.

Efficacy was measured in two ways after at least 15 minutes of constant stimulation at the previously determined efficacy threshold for each setting: UPDRS III evaluation (including lateralized scores calculated by summing items for the left and right side separately), and an objective, quantitative assessment of tremor and bradykinesia using a finger-worn motion sensor (Kinesia ProView, Great Lakes Neurotechnologies). In the latter assessment, patients performed three tasks while wearing a motion sensor on the tip of the index finger: rest tremor assessment, a finger tapping task, and rapidly alternating movement task.

Results. Sixteen subjects were enrolled in the study. One subject did not tolerate reprogramming and withdrew from the study before any data could be collected.

For the 15 subjects that completed the study, current steering to the adjacent dorsal or ventral electrode resulted in little change in mean therapeutic window (mean therapeutic window = 2.56 mA (dorsal current steering) vs. 2.05 mA (ventral current steering) vs. 2.32 mA (best therapeutic contact) (Figure 1). However, a trend was observed towards higher thresholds for both efficacy and side effects when using current steering in the dorsal direction (side effect threshold = 5.68 mA (dorsal) vs. 4.63 mA (best therapeutic electrode) vs. 4.56 mA (ventral electrode); efficacy threshold = 3.12 mA (dorsal) vs. 2.31 mA (best therapeutic electrode) vs. 2.51 mA (ventral) (Figure 1).

The total charge delivered per pulse at efficacy threshold was slightly higher using current steering settings, particularly in the case of dorsal steering (138·4 nC/pulse at best therapeutic electrode vs. 150.6 nC/pulse (ventral) and 187.2 nC/pulse (dorsal)). This is consistent with the trend towards higher thresholds when using current steering in the dorsal direction, while keeping pulse width constant at 60 µs.

Figure 1. Changing the location of stimulation using dorsal or ventral current steering, while keeping pulse width constant, had little or no effect on mean therapeutic window, but did result in higher mean thresholds in the dorsal direction.

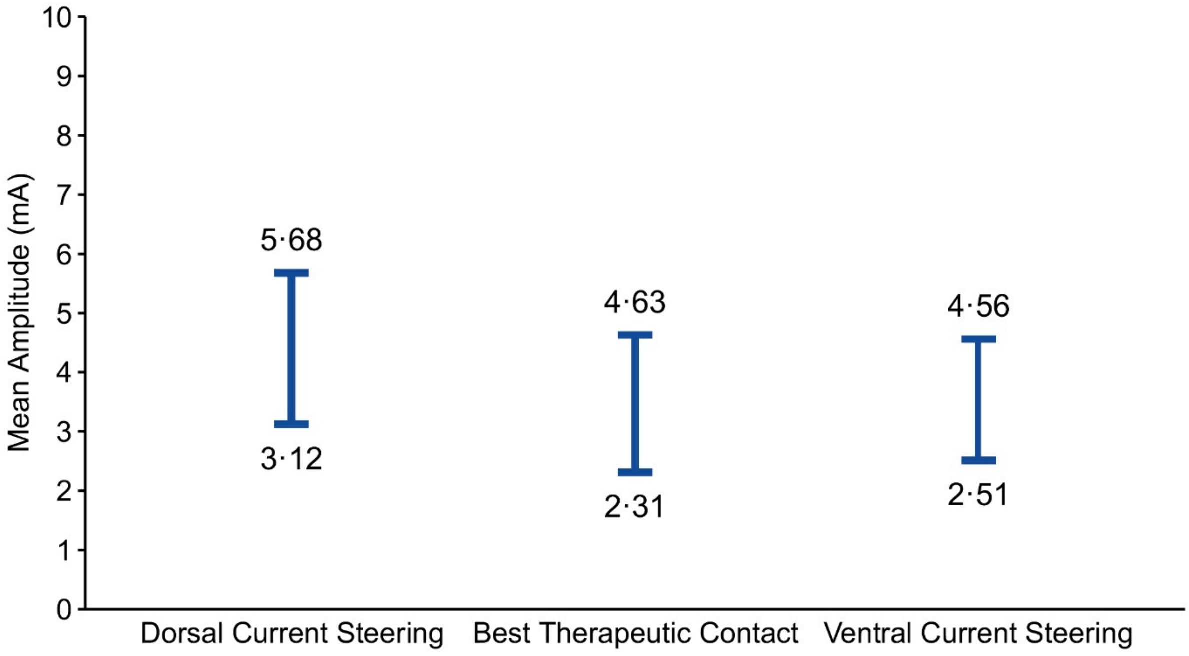

Discussion. This is the first study to measure an effect size of current steering on efficacy and side effect thresholds. Our preliminary exploration of current steering was constrained by the time limitations of a programming visit, but after testing only two locations in the vicinity of the best therapeutic electrode, we were able to observe effects of small changes in the location of stimulation on efficacy and side effect thresholds. These small changes in thresholds suggest that current steering enables adjustments of stimulation that may be useful for improving the therapeutic response or avoiding a side effect in some patients. The result is more difficult to test statistically, since the location of the electrode relative to STN varies from side to side as well as between patients, and therefore the optimal location of stimulation relative to the electrode is also expected to vary. While individual patients displayed differences in therapeutic window at different current steering locations, the mean therapeutic window across all patients showed little change. A more thorough examination of current steering which accounts for this variability among patients is the focus of a follow-up study.
